# Correction: Posttraumatic Stress Disorder Increases Sensitivity to Long Term Losses among Patients with Major Depressive Disorder

**DOI:** 10.1371/journal.pone.0095819

**Published:** 2014-04-16

**Authors:** The PLOS ONE Staff

## Notice of Republication

This article was republished on March 13 2014, to include the supplementary information (Figure S1, Table S1-S6) that were corrupt in the original version. Please download this article again to view the correct version. The originally published, uncorrected article and the republished, corrected article are provided here for reference.

## Supporting Information

File S1 Originally published, uncorrected article.
(PDF)

File S2 Republished corrected article.
(PDF)
