# Supplementary material for: Posttraumatic Stress Disorder Increases Sensitivity to Long Term Losses among Patients with Major Depressive Disorder
Source: PLoS One. 2013 Oct 7;8(10):e78292. doi: 10.1371/journal.pone.0078292 (PMC3792061; doi:10.1371/journal.pone.0078292)
Supplement: File S1 — Text S1, Text S2, Figure S1, Tables S1-S6. Text S1. Description of Additional Questionnaires. Text S2. Model Selection. Figure S1. Frequencies of selecting the safe choice option during the risk task in all trials (A) and after punishment only (B) across three groups. The risk task was analyzed using both two (results reported in main paper) and three groups, in order to account for the moderating effects of comorbid PTSD. Results for the three-group analysis investigating the moderating effect of comorbid PTSD replicated findings for the two-group analysis. No difference between the groups were found for choice frequencies of safe option overall (A) and after punishment (B). Specifically, as illustrated in the box plots risky choice behavior did not differ between the HC, MDD-only and MDD+PTSD groups [Safe choice shown in A: X2 (df = 2, N = 36) = 2.2973, p = 0.3171; Safe choice after punishment shown in B: X2 (df = 2, N = 36) = 1.731, p = 0.421]. Table S1. Model comparison results across three commonly used discounting functions for the combined MDD analysis (see Text S2). The winning model is highlighted in bold. Table S2. Model comparison results across three commonly used discounting functions when stratifying the MDD group by comorbid PTSD diagnosis (see Text S2). The winning model is highlighted in bold. Table S3. Model comparison results across three commonly used discounting functions for control subjects (see Text S2). The winning model is highlighted in bold. Table S4. Model comparison results across three commonly used discounting functions for all MDD subjects (see Text S2). The winning model is highlighted in bold. Table S5. Model comparison results across three commonly used discounting functions for MDD only subjects (see Text S2). The winning model is highlighted in bold. Table S6. Model comparison results across three commonly used discounting functions for MDD+PTSD subjects (see Text S2). The winning model is highlighted in bold. (PDF) [file pone.0078292.s001.pdf]

Supplemental Material for
Primary Posttraumatic Stress Disorder Increases Sensitivity to Long Term Losses among Patients with Major Depressive Disorder

Jan B. Engelmann*, Britta Maciuba*, Christopher Vaughan, Martin Paulus, Boadie W. Dunlop

1 Description of Additional Questionnaires

Barrat Impulsiveness Scale 11 (BIS-11).
The BIS-11 is a widely used measure of self-reported impulsivity, with higher scores reflecting greater impulsivity. In addition to an overall measure, the BIS provides three factors assessing different forms of impulsivity. Attentional Impulsivity reflects the difficulty focusing on the task at hand and experiencing racing or intrusive thoughts. Motor Impulsivity consists of actions taken without forethought and a lack of perseverance or inconsistent lifestyle. Non-planning Impulsivity is characterized by difficulties in planning and ordered thinking, along with discomfort with complex mental tasks.
Flinders Decision-Making Questionnaire (FDMQ).
The FDMQ provides data on four different qualities of decision-making. Vigilance is considered a healthy, rational decision-making style, in which goals are clarified, data gathered and alternatives considered carefully. Hypervigilance reflects frantic efforts to solve problems, due to a sense of time pressure or impulsivity. Buck-passing reflects a form of avoidance in decision-making manifested by deferring to others and avoidance of responsibility. Procrastination is another form of decision-making avoidance characterized by delay and passive neglect of issues requiring choices.

2 Model Selection 1: all data

We compared three general classes of discounting functions commonly employed in experimental economics to model intertemporal choice behavior (e.g., Berns, Laibson, & Loewenstein, 2007; Kable & Glimcher, 2007). Furthermore, for each model class, we compared full models to their reduced versions, for which specific parameters were deleted. Model comparison was done separately for two potential groupings of depressed subjects, namely once comparing all depressed subjects to healthy controls (results are shown in Table 1) and once after further stratifying the depressed group based on the presence of absence of comorbid PTSD (results are shown in Table 2). The following model classes were included in model comparisons:
1. a standard exponential model:

$$SV = e^{-cD} \tag{1}$$

where SV is subjective value expressed as a fraction of immediate value, c is the exponential discounting parameter and D is the delay in years.
2. a standard hyperbolic model:

$$SV = \frac{1}{1 + kD} \tag{2}$$

where k is the hyperbolic discounting factor and D is the delay in years.
3. a quasi-hyperbolic model:

$$SV = \frac{(e^{-\beta D} + e^{-\delta D})}{2} \tag{3}$$

where β reflects a special weight placed on more immediate rewards and δ is reflective of more consistent weighting of time periods (e.g., Kable & Glimcher, 2007; McClure, Laibson, Loewenstein, & Cohen, 2004). Full models within each of the three model types were compared against their reduced counterparts, in which a number of parameters and combinations of these were systematically removed, as indicated in Table 1. Full models included parameters to estimate the effect of loss vs. gain framing and patient group on discounting. Since the goal of estimations was to assess group differences in discounting, parameters estimating group differences in discounting slope were included in all models, but reduced models did not include additional parameters distinguishing between loss and gain frames.
Model selection was conducted using Akaike weights, w_i, based on corrected AIC values as recommended by various authors (Wagenmakers & Farrell, 2004; Burnham & Anderson, 2002). The AIC was converted to a corrected AIC by adding the term 2 k * (k + 1) / (n - k - 1), where k is the number of parameters and n is the number of observations (Wagenmakers & Farrell, 2004; Burnham & Anderson, 2002). Akaike weights reflect the probability that a given model, M_i, is the best model among the alternative models given the data. Akaike weights were computed by first obtaining an estimate of the relative likelihood of the model and then dividing this term by the sum of all model likelihoods as follows:

$$w_i(AICc) = \frac{e^{-0.5(AICc_i - min(AICc))}}{\sum_{k=1}^{k} e^{-0.5(AICc_i - min(AICc))}} \tag{4}$$

Based on Akaike weights, quasi-hyperbolic models were found to provide superior fits to the data despite controlling for model complexity and independent of grouping of depressed subjects. Model comparisons also show the 6- and 9-parameter quasi-hyperbolic model, depending on whether MDD subjects were stratified or not, to be the clear winner against all other full and reduced models. In fact, relative to the next best model, the 6- (9-) parameter quasi-hyperbolic model is 6.19 (2.92) times more likely to be the best model in terms of the Kullback-Leibler discrepancy. Results from the 6- and 9-parameter quasi-hyperbolic models are therefore reported in the main paper. The winning models take the following form:
1. for the combined MDD analysis:

$$SV = \frac{(e^{-(\beta_1+\beta_2 g_1)D} + e^{-(\delta_1+\delta_2 l+\delta_3 g_1+\delta_4(l g_1))D})}{2} \tag{5}$$

2. when further stratifying MDD patients by PTSD diagnosis:

$$SV = \frac{(e^{-(\beta_1+\beta_2 g_1+\beta_3 g_2)D} + e^{-(\delta_1+\delta_2 l+\delta_3 g_1+\delta_4 g_2+\delta_5(l g_1)+\delta_6(l g_2))D})}{2} \tag{6}$$

where β reflects early discounting and δ reflects late discounting, g_1 and g_2 are dummies employed to assess group differences in discounting slopes and l is a dummy employed to assess slope differences between gain and loss frames via multiplication with relevant parameters.

Table S 1: Model comparison results across three commonly used discounting functions for the combined MDD analysis

	Model Form	Model Complex.	Number Params.	AICc	Δ_i	Relative Likelih.	Akaike w_i	Log Likelih.	Least Sq. Residuals
1	exponential	full	4.00	148.70	97.85	0.00	0.00	-69.30	-27.84
2	exponential	reduced	2.00	164.59	113.75	0.00	0.00	-79.28	-30.34
3	hyperbolic	full	4.00	109.71	58.86	0.00	0.00	-49.81	-17.50
4	hyperbolic	reduced	2.00	124.26	73.42	0.00	0.00	-59.12	-17.64
5	quasi-hyperbolic	full	8.00	54.49	3.65	0.16	0.13	-18.07	-2.87
6	quasi-hyperbolic	reduced	6.00	50.84	0.00	1.00	0.83	-18.32	-3.31
7	quasi-hyperbolic	reduced	5.00	57.45	6.61	0.04	0.03	-22.66	-2.98

Table S 2: Model comparison results across three commonly used discounting functions when stratifying MDD group by comorbid PTSD diagnosis

	Model Form	Model Complex.	Number Params.	AICc	Δ_i	Relative Likelih.	Akaike w_i	Log Likelih.	Least Sq. Residuals
1	exponential	full	6.00	144.74	96.65	0.00	0.00	-65.27	-27.40
2	exponential	reduced	3.00	165.58	117.49	0.00	0.00	-78.76	-30.41
3	hyperbolic	full	6.00	103.66	55.56	0.00	0.00	-44.73	-16.78
4	hyperbolic	reduced	3.00	125.74	77.65	0.00	0.00	-58.84	-17.80
5	quasi-hyperb.	full	12.00	50.21	2.12	0.35	0.24	-11.72	-2.96
6	quasi-hyperb.	reduced	9.00	48.09	0.00	1.00	0.70	-13.83	-3.13
7	quasi-hyperb.	reduced	7.00	53.20	5.11	0.08	0.05	-18.46	7.00

3 Model Selection 2: groupwise data

The possibility that behavior of different groups (HC, MDD, MDD+PTSD) is best fitted by different discounting models was tested. To this end, we performed model selection at the group level. We followed the same logic as for the model selection procedure outlined in section 2, for which we used all available data and modeled group influence via specific parameters. However, we only used limited data, such that model selection was performed for each group separately. Results strengthen the conclusion from section 2 and underline that quasi-hyperbolic models best describe the data, even when assessed at the group-level.

Table S 3: Model comparison results across three commonly used discounting functions for Control subjects

	Model Form	Model Complex.	Number Params.	AICc	Δ_i	Relative Likelih.	Akaike w_i	Log Likelih.	Least Sq. Residuals
1	exponential	full	2.00	32.26	33.00	0.00	0.00	-13.10	-10.36
2	exponential	reduced	1.00	35.56	36.30	0.00	0.00	-15.77	-10.94
3	hyperbolic	full	2.00	21.37	22.11	0.00	0.00	-7.65	-7.15
4	hyperbolic	reduced	1.00	25.47	26.21	0.00	0.00	-10.72	-7.63
5	quasi-hyperbolic	full	4.00	0.81	1.55	0.46	0.32	4.71	-0.49
6	quasi-hyperbolic	reduced	3.00	-0.74	0.00	1.00	0.68	4.44	-0.89

Table S 4: Model comparison results across three commonly used discounting functions for all MDD subjects

	Model Form	Model Complex.	Number Params.	AICc	Δ_i	Relative Likelih.	Akaike w_i	Log Likelih.	Least Sq. Residuals
1	exponential	full	2.00	113.81	62.83	0.00	0.00	-53.88	-17.48
2	exponential	reduced	1.00	125.52	74.54	0.00	0.00	-60.75	-19.39
3	hyperbolic	full	2.00	87.12	36.14	0.00	0.00	-40.54	-10.35
4	hyperbolic	reduced	1.00	97.15	46.17	0.00	0.00	-46.57	-10.00
5	quasi-hyperbolic	full	4.00	53.03	2.05	0.36	0.26	-21.43	-2.38
6	quasi-hyperbolic	reduced	3.00	50.98	0.00	1.00	0.74	-21.44	-2.42

Table S 5: Model comparison results across three commonly used discounting functions for MDD only subjects

	Model Form	Model Complex.	Number Params.	AICc	Δ_i	Relative Likelih.	Akaike w_i	Log Likelih.	Least Sq. Residuals
1	exponential	full	2.00	58.17	29.80	0.00	0.00	-26.04	-8.82
2	exponential	reduced	1.00	57.52	29.15	0.00	0.00	-26.74	-9.12
3	hyperbolic	full	2.00	43.19	14.82	0.00	0.00	-18.55	-4.65
4	hyperbolic	reduced	1.00	41.58	13.21	0.00	0.00	-18.78	-4.46
5	quasi-hyperbolic	full	4.00	28.72	0.35	0.84	0.46	-9.20	-1.03
6	quasi-hyperbolic	reduced	3.00	28.37	0.00	1.00	0.54	-10.09	-1.14

Table S 6: Model comparison results across three commonly used discounting functions for MDD+PTSD subjects

	Model Form	Model Complex.	Number Params.	AICc	Δ_i	Relative Likelih.	Akaike w_i	Log Likelih.	Least Sq. Residuals
1	exponential	full	2.00	54.51	31.85	0.00	0.00	-24.20	-8.22
2	exponential	reduced	1.00	69.89	47.23	0.00	0.00	-32.93	-10.34
3	hyperbolic	full	2.00	40.79	18.13	0.00	0.00	-17.34	-4.98
4	hyperbolic	reduced	1.00	57.76	35.10	0.00	0.00	-26.86	-5.70
5	quasi-hyperbolic	full	4.00	23.14	0.48	0.79	0.44	-6.38	-1.44
6	quasi-hyperbolic	reduced	3.00	22.66	0.00	1.00	0.56	-7.21	-1.10

4 Risk task results across three groups

The risk task was analyzed using both two (reported in main paper) and three groups, in order to account for the moderating effects of comorbid PTSD. Results for the three-group analysis investigating the moderating effect of comorbid PTSD replicated findings for the two-group analysis. No difference between the groups were found for choice frequencies of safe option overall and after punishment. Specifically, risky choice behavior did not differ between the HC, MDD-only and MDD+PTSD groups [Safe choice: X2 (df = 2, N = 36) = 2.2973, p = 0.3171; Safe choice after punishment: X2 (df = 2, N = 36) = 1.731, p = 0.421].

Figure S 1: Choice frequencies during the risk task across three groups

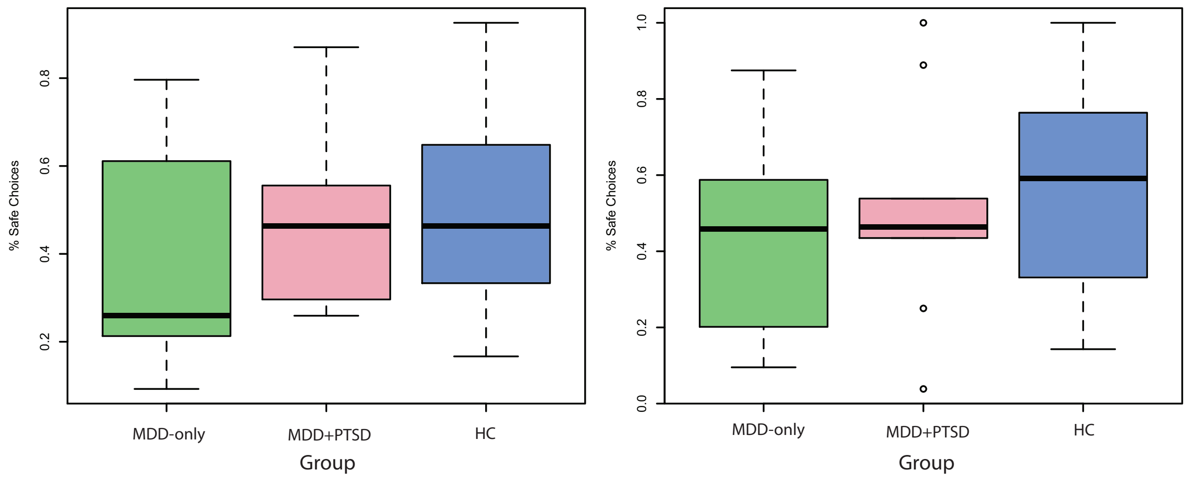

References

Berns, G., Laibson, D., & Loewenstein, G. (2007). Intertemporal choice-toward an integrative framework. *Trends in Cognitive Sciences*, *11*, 482–488.

Burnham, K. P., & Anderson, D. (2002). *Model selection and multi-model inference* (2nd edition ed.). Springer.

Kable, J. W., & Glimcher, P. W. (2007, December). The neural correlates of subjective value during intertemporal choice. *Nature neuroscience*, *10*(12), 1625–1633.

McClure, S. M., Laibson, D. I., Loewenstein, G., & Cohen, J. D. (2004, October). Separate neural systems value immediate and delayed monetary rewards. *Science (New York, N.Y.)*, *306*(5695), 503–507.

Wagenmakers, E.-J., & Farrell, S. (2004, February). AIC model selection using Akaike weights. *Psychonomic Bulletin & Review*, *11*(1), 192–196.
