# Supplementary material for: Correction: Posttraumatic Stress Disorder Increases Sensitivity to Long Term Losses among Patients with Major Depressive Disorder
Source: PLoS One. 2014 Apr 16;9(4):e95819. doi: 10.1371/journal.pone.0095819 (PMC3989339; doi:10.1371/journal.pone.0095819)
Supplement: File S1 — Originally published, uncorrected article. (PDF) [file pone.0095819.s001.pdf]

Posttraumatic Stress Disorder Increases Sensitivity to Long Term Losses among Patients with Major Depressive Disorder

Jan B. Engelmann[1☯], Britta Maciuba[2☯], Christopher Vaughan[3], Martin P. Paulus[4], Boadie W. Dunlop[3*]

1 Department of Economics, University of Zurich, Zürich, Switzerland, **2** Department of Family Medicine, Scott and White Hospital, Texas A&M Health Center, Temple, Texas, United States of America, **3** Mood and Anxiety Disorders Program, Department of Psychiatry and Behavioral Sciences, Emory University School of Medicine, Atlanta, Georgia, United States of America, **4** Department of Psychiatry, University of California San Diego, La Jolla, California, United States of America

Abstract

Background: Decisions under risk and with outcomes that are delayed in time are ubiquitous in real life and can have a significant impact on the health and wealth of the decision-maker. Despite its potential relevance for real-world choices, the degree of aberrant risky and intertemporal decision-making in patients suffering from major depressive disorder (MDD) and posttraumatic stress disorder (PTSD) has received little attention to date.

Method: We used a case-control design to compare decision-making in healthy control subjects (N=16) versus untreated depressed subjects in a current major depressive episode (N=20). In order to examine how major depressive disorder (MDD) may impact decision-making, subjects made decisions over (1) risky outcomes and (2) delayed outcomes in the domain of gains and losses using choice paradigms from neuroeconomics. In a pre-planned analysis, depressed subjects were subdivided into those with primary PTSD along with comorbid MDD (MDD+PTSD) versus those with primary MDD without PTSD (MDD-only). Choice behavior was modeled via a standard econometric model of intertemporal choice, a quasi-hyperbolic temporal discounting function, which was estimated for each subject group separately.

Results: Under conditions of potential gain, depressed subjects demonstrated greater discounting for gains across all time frames compared to controls. In the realm of losses, both subgroups of depressed subjects discounted more steeply than controls for short time frames. However, for delayed losses ranging from >1-10 years, MDD+PTSD subjects showed shallower discounting rates relative to MDD-only subjects, who continued to discount future losses steeply. Risk attitudes did not contribute to differences in intertemporal choice.

Conclusions: Depressed patients make choices that minimize current pain and maximize current reward, despite severe later consequences or lost opportunities. Anxiety associated with PTSD may serve as a partially protective factor in decision-making about long-term potential losses compared to MDD patients without PTSD.

Citation: Engelmann JB, Maciuba B, Vaughan C, Paulus MP, Dunlop BW (2013) Posttraumatic Stress Disorder Increases Sensitivity to Long Term Losses among Patients with Major Depressive Disorder. PLoS ONE 8(10): e78292. doi:10.1371/journal.pone.0078292

Editor: Amanda Bruce, University of Missouri-Kansas City, United States of America

Received April 18, 2013; **Accepted** July 30, 2013; **Published** October 7, 2013

Copyright: © 2013 Engelmann et al. This is an open-access article distributed under the terms of the Creative Commons Attribution License, which permits unrestricted use, distribution, and reproduction in any medium, provided the original author and source are credited.

Funding: This work was supported by a National Institutes of Mental Health grant, K23 MH086690 (BWD). JBE gratefully acknowledges support from the Mercator Foundation Switzerland, the NCCR Affective Sciences, the Neurochoice project of SystemsX and the research priority program at the University of Zurich 'Foundations of Human Social Behavior'. The funders had no role in study design, data collection and analysis, decision to publish, or preparation of the manuscript.

Competing interests: The authors have declared that no competing interests exist.

* E-mail: bdunlop@emory.edu

☯ These authors contributed equally to this work.

Introduction

Major depressive disorder (MDD) is a chronic and debilitating disease with a lifetime prevalence of 13-17% [1,2]. In addition to sad mood and loss of interest in activities, MDD is frequently associated with pessimism about future possibilities and reduced sensitivity to rewards [3,4].

Posttraumatic stress disorder (PTSD) affects 2-7% of the population [1,2]. PTSD is classified in DSM-IV as an anxiety disorder that shares considerable symptom overlap with MDD, including insomnia, loss of interests, and concentration impairments. MDD frequently co-occurs in patients with PTSD, leading some to question whether PTSD is truly a distinct illness [5], though others find support for the current DSM-IV

classification [6,7]. Among the features of PTSD that distinguish it from MDD are distress from, and avoidance of, reminders of the traumatic event, hyper-vigilance for threat, and a foreshortened sense of one's future [8].

An important but understudied clinical issue for patients suffering from depression is decision-making behavior. Depressed patients often make choices that are not in their best interest, such as neglect of social relationships, reacting passively to potential defeats, and failing to pursue new opportunities [9.10]. Such choices can serve to continue or intensify the external circumstances that act to reinforce the negative mood state. Furthermore, depressed patients may look to use short-term external environmental factors to improve their mood, choosing to pursue immediate rewards to the neglect of long-term benefits or costs. This decision-making style is consistent with observations that depressed patients may overeat, abuse substances, or shop excessively as efforts at mood repair [11,12], despite the long-term negative consequences of these behaviors. Patients with PTSD often make choices demonstrating excessive concern about potential threat and vulnerability, leading to behaviors that are avoidant or that reflect marked fear and expectation of negative future events, often experienced as a sense of dread.

Neuroimaging studies have identified alterations in the brains of patients with MDD and PTSD. Structurally, both MDD and PTSD patients demonstrate reduced volumes in anterior cingulate cortex, hippocampus and thalamus relative to healthy controls [13]. Findings present in MDD, but not PTSD, patients include reduced orbitofrontal cortex (OFC) volume, while PTSD patients show decreased inferior temporal cortex and amygdala volumes more consistently than MDD patients [14,15]. From functional imaging the most consistent finding in MDD patients is heightened resting state activity compared to controls in subgenual and pregenual anterior cingulate cortex [16]. Hypoactivity in dorsolateral prefrontal cortex (DLPFC), a region frequently implicated in executive control [17], is another common finding [18]. In PTSD, the best replicated finding is hyperactivation of the amygdala, a region implicated in fear processing and salience detection [19,20], and reduced activation of the medial PFC in response to potential threat stimuli [21,22]. Experienced reward is processed via signaling in the striatum, and hypoactivity in these regions compared to controls have been reported for both MDD [23] and PTSD [24].

Several of these brain regions affected in MDD and PTSD also serve as important processing areas within networks associated with valuation and decision-making [25]. Intertemporal choice is one of the most ubiquitous forms of decision making, the outcomes of which can significantly impact individuals' personal and professional success, including their health and wealth [26]. Intertemporal decisions involve temporally delayed consequences that involve tradeoffs between immediate consumption and planning for a better outcome. Temporal discounting (also known as delay discounting) refers to the well-established tendency in animals and humans to place less value on a reward or cost as the time to receipt of that reward or cost moves further into the future [27,28]. Delay discounting in psychiatrically healthy individuals typically displays a quasi-hyperbolic discounting function, showing steep declines in the perceived value of a reward in the relatively near future, with a flattening of the decline over more distant time periods [29]. Discounting for delayed losses is usually less steep than for delayed gains [30,31], indicating that for most people, large future losses are more aversive than large future gains are attractive.

In psychiatry, greater discounting of future rewards has been most consistently demonstrated for patients with addictive disorders [32,33]. Whether the established clinical and neurobiological differences between MDD and PTSD are associated with differences in delay discounting has not been determined. In a sample of healthy college students, higher levels of self-reported anhedonic symptoms were correlated with less steep discounting of future gains, suggesting that non-clinical levels of anhedonia may be associated with greater willingness to wait for a larger delayed reward compared to a smaller reward received immediately [34]. In another study, healthy subjects exposed to a tryptophan-depleted condition (which results in lower availability of central nervous system serotonin) more steeply discounted the value of future gains than non-depleted subjects [35]. Only one prior published study has examined temporal discounting in a clinical sample of patients experiencing a current major depressive episode. In a mixed sample of Japanese unipolar and bipolar depressed patients, Takahashi and colleagues found depressed patients discounted both future gains and future losses less steeply than controls [36]. No previous work has examined these decision-making variables in PTSD patients.

In the current study, we compared intertemporal decision-making in healthy controls with subjects currently experiencing a major depressive episode. Furthermore, we assessed whether decision-making among MDD patients differed between subjects for whom MDD was the primary illness (MDD-only) versus those for whom MDD was secondary to a primary diagnosis of PTSD (MDD+PTSD). Specifically, we explored: 1) whether there are differences in risk-taking and temporal discounting between depressed subjects and psychiatrically healthy subjects; and 2) whether MDD-only subjects differ in their choice behavior from MDD+PTSD subjects in the context of choice scenarios probing risk and intertemporal economic preferences. Because differences in risk attitudes may affect intertemporal choice behavior [37,38], these two components of decision-making should be simultaneously assessed, though this has not been done in previous studies of intertemporal choice in patients. We expected depressed subjects to show greater risk-taking and steeper temporal discounting curves than healthy control participants. We also hypothesized that MDD+PTSD subjects would show steeper temporal discounting for gains and losses than pure MDD subjects.

Materials and Methods

Study Design

We used a case-control design to compare subjects with depression and healthy, non-depressed control subjects on factors related to decision-making. The Emory Institutional Review Board reviewed and approved the study protocol. The

study was conducted in accord with the Declaration of Helsinki and its amendments.

Participants

Participants were recruited from ongoing studies of MDD and PTSD in the Mood and Anxiety Disorders Program of Emory University and from flyers posted on the Emory University campus. All subjects were English speakers between the ages of 18-65 years and signed a written informed consent form to participate.

To be eligible, depressed subjects had to meet criteria for MDD and be in an active major depressive episode diagnosed using the Structured Clinical Interview for DSM-IV (SCID) [39], and score ≥18 on the Hamilton Rating Scale for Depression (HAM-D) for the past week, reflecting at least a moderate level of depression severity [40]. Subjects with MDD were divided into two subgroups: 1) those who had a primary diagnosis of MDD and who did not meet criteria for PTSD were classified as the MDD-only group; 2) those who met SCID criteria for PTSD, and for whom PTSD was the primary diagnosis, were classified as the MDD+PTSD group. Eligibility criteria for healthy controls (HC) included an absence of any current Axis I psychiatric disorder, absence of lifetime history of MDD, and a past-week HAM-D score ≤7, the standard cut-point for that scale representing minimal to absent depressive symptoms. All participants had to report being free of psychotropic medications, as well as herbal or other over-the-counter preparations with psychotropic effects, for one week prior to testing.

Exclusionary criteria for all subjects included a lifetime diagnosis of bipolar disorder or a psychotic disorder, acute clinically significant suicide risk, substance abuse or dependence in the previous three months, or active central nervous system-related illness that would affect ability to complete the test procedures.

Measures

Anxiety was assessed with the Hamilton Anxiety Rating Scale (HAM-A) [41]. Self-reported adverse childhood experiences were measured with the Childhood Trauma Questionnaire (CTQ) [42]. To assess personality factors related to decision-making, two standard self-report questionnaires were administered: the Barratt Impulsiveness Scale Version 11 (BIS), which determines an overall measure of impulsivity, along with three factors: attentional, non-planning, and motor impulsivity [43], and the Flinders Decision-Making Questionnaire (FDMQ) [44], which provides data on four different qualities of decision-making. These qualities include Vigilance, which reflects a healthy, rational decision-making style, along with Hypervigilance, Buck-passing and Procrastination, which reflect maladaptive problem-solving styles. See supplemental materials (Text S1 in File S1) for a more detailed description of the BIS-11 and FDMQ.

Decision-making Tasks

After completion of the clinician interviews and self-report questionnaires, subjects completed a series of standard computerized decision-making tasks, using either points or hypothetical monetary payouts as outcomes.

In the Risky Gains task [45], subjects make decisions about whether to accept a smaller amount of points at no risk, or whether to wait for increasing amounts, but at a higher risk. The goal of this task is to collect as many points as possible. Subjects view a series of increasing numbers (20, 40, 80), which remain on screen for 1 second. Points can be collected by accepting the number shown on the screen at a given moment via button press. Accepting the 20-point option is always a safe bet; however, waiting for higher value choice options can lead to an immediate loss of all points on that trial and therefore carries risk. Trials in which subjects can lose points are referred to as “punished” trials. There are 96 trials presented randomly (54 unpunished, 24 punished-40, 18 punished-80). The task assesses two behaviors related to the relative frequency of risky choices (1) over all trials, reflective of baseline risk attitude and (2) after punishment was received, reflective of punishment sensitivity.

In the Delay Discounting Task [46], subjects are instructed to make binary choices between smaller but sooner and later but larger hypothetical monetary payouts (dollars). The subjects make repeated choices (prefer immediate reward or delayed future reward) for each of six future time points. For each time point, a “delay equivalent” was determined using a standard bisection method. Delay equivalents are the indifference points between an immediate and delayed outcome and reflect the subjective value that subjects place on an outcome that occurs in the future. The bisection method followed the following algorithm: For each delay, there were eight choice trials, in which the future reward amount was fixed, and the amount of the immediate reward was varied. Amounts of immediate gains for a given delayed choice option were adjusted based on subjects’ prior choices, such that the immediate gain magnitude offered on the current trial was increased by stepsize δ when the subject chose the delayed amount on the prior trial and decreased by δ when the immediate gain was chosen. Specifically, the range between 0 and the fixed delayed reward value was divided into thirds. The one-third and two-third cut point amounts were presented as the immediate reward option on the first two choice trials. This procedure identified which third of the range would be used in the subsequent choice trials for that time point. The magnitude of the adjustment parameter, δ, was decreased on each trial until a best estimate of the decision equivalent was reached after 8 trials (see 43 for a similar procedure over risky choice options). This procedure was repeated for each of six delayed gains, with delay d, in days, months, or years and a fixed gain amount, x in dollars, which consisted of (d, x) = (5y, 10360), (30d, 10260), (10y, 9740), (3y, 9880), (1y, 9640), (6m, 10120). The reward amount, x, was varied slightly to avoid stereotyped responding. The task was repeated with equivalent parameters for losses, such that subjects chose whether they preferred an immediate loss now, or a future greater loss. The following six delayed losses were included (d, x) = (6m, 9740), (6y, 9880), (30d, 9640), (5y, 10120), (10y, 10360), (1y, 10260).

Statistical Analysis

Initial analyses compared responses of HC and the depressed groups to assess questionnaire and behavioral differences between the two groups. In a pre-planned analysis, we repeated all comparisons using the three groups (HC, MDD-only, and MDD+PTSD) to examine the moderating effects of primary PTSD within the depressed group. For the risky gains task, the percentage of risky choices overall and after punishment was compared across subject groups using nonparametric tests. Specifically, for comparisons of two groups, the two-sample Wilcoxon test was employed, while for comparisons of three groups, the Kruskal-Wallis test was used for initial group comparisons and, if significant differences were identified, follow-up analyses were conducted using the two-sample Wilcoxon test. Repeating all non-parametric analyses using the equivalent parametric approach (one-way between-subjects ANOVA and independent samples t-test) identified the same trends.

For the Delay Discounting task, subjective values reflective of the indifference point between the choice options offering immediate and delayed rewards were entered as response variables into a standard econometric model [47,48]. Specifically, a quasi-hyperbolic discounting function was fit to the data to assess the influence of framing (gain vs. loss) and group on discounting slopes for choice options in the near and distant future. The quasi-hyperbolic model was selected via a rigorous model selection procedure, in which we compared three standard discounting functions commonly employed to model intertemporal choice behavior, namely full and reduced forms of an exponential, a hyperbolic, and a quasi-hyperbolic model. We performed model selection on the aggregate data (see Text S2 in File S1 for a detailed description of the model selection procedure and Tables S1 and S2 in File S1 for results), as well as on each group (see Tables S3-S6 in File S1 for results). In both cases the reduced quasi-hyperbolic model was clearly superior. See supplemental materials (Text S2 in File S1) for model selection results and detailed description of the selected models.

Non-linear least squares regression implemented in R (http://cran.us.r-project.org) was employed to assess differences in temporal discounting slopes for gains and losses as a function of group. The use of a quasi-hyperbolic model allowed us to estimate group-specific parameters reflective of discounting slopes for early (β, delays < 1) and late (δ, delays of >1-10) choice options as well as for gains and losses. Our model extracted parameters for representative subjects that were clustered based on their diagnosis. Using aggregate data to estimate behavioral parameters based on choices in economic games and choice tasks is a standard econometric approach in behavioral economics as well as neuroeconomics [49-52]. We conducted nonparametric bootstrap analyses on parameter estimates from our best fitting model using the boot package implemented in R (N=35, resampling with replacement, ca. 25,000 replications) [53]. Because our aggregate model pools data from various subjects with the same diagnosis, resampling was stratified based on subject to correct for non-independence of choices. Bias-corrected and accelerated two-tailed 95% confidence intervals for parameter estimates were calculated.

Results

Thirty-six participants consented to participate in the study: 20 depressed subjects (MDD-only=11, MDD+PTSD=9) and 16 HC. Data from one HC could not be extracted and was excluded from the Delay Discounting Task analysis. Demographic data for the sample are presented in Table 1. Minorities (7 African-American, 2 Asian, and 6 multiracial) comprised 42% of the sample. There were no significant differences found between the HC and depressed groups for any of the demographic variables.

Table 1 also presents the differences between the depressed and HC participants on the depression, anxiety and childhood trauma measures. As expected, depression severity as assessed by HAM-D scores was significantly lower in HC than in the depressed subjects; depression severity was similar between the MDD-only and MDD+PTSD groups. MDD+PTSD subjects were significantly more anxious than MDD-only subjects. Both MDD-only and MDD+PTSD subjects were significantly more likely to have suffered childhood trauma than HC subjects based on the CTQ total score, and MDD+PTSD subjects reported significantly more childhood trauma than MDD-only subjects.

Risky Gains Task

As shown in Figure 1, depressed subjects did not make riskier choices (i.e., choosing the "risky" 40 or 80 point value versus the "safe" 20 point value) than HC [$W = 134.5$, $p = 0.426$, $r = 0.135$]. Depressed and HC subjects also showed no significant difference in whether they chose a "safe" 20 point option or a "risky" 40 or 80 point option after a round in which they were punished and lost points [$W = 122$, $p = 0.233$, $r = 0.202$]. The three-group analysis reproduced the results from the two-group analysis (Figure S1 in File S1). Specifically, risky choice behavior did not differ between the HC, MDD-only and MDD+PTSD groups [Safe choice: X^2 (df = 2, N = 36) = 2.2973, $p = 0.3171$; or safe choice after punishment: X^2 (df = 2, N = 36) = 1.731, $p = 0.421$]. These results indicate that the presence of depression (with or without PTSD) did not differentially impact choice frequencies over options involving risks over hypothetical outcomes.

Temporal Discounting Task

General effects of depression on intertemporal decision-making. Figure 2A illustrates the results from the best-fitting 6-parameter quasi-hyperbolic model employed to examine general effects of depression on intertemporal decision-making. Table 2 shows parameter estimates for depressed and healthy control groups that reflect slopes for early discounting for delays up to 1 year (β), and late discounting for both gains (δ) and losses ($\delta * l$) for delays greater than 1 year. Of greatest significance to the current analyses are parameter estimates reflective of comparisons between the depressed and healthy control groups, including group effects on (1) early discounting ($\beta * g$), (2) late discounting over gains ($\delta * g$) and (3) late

Table 1. Demographic and clinical characteristics of depressed and healthy control subjects.

Characteristic	HC (N=16)	Depressed (N=20)	p-value	Pair-wise Comparison	p-value
AGE, mean (SD), y	35.1 (16.0)	38.6 (12.6)	0.202	N/A	N/A
GENDER			0.647	"	"
Female (%)	12 (75)	15 (75)		"	"
Male (%)	4 (25)	5 (25)		"	"
RACE			0.214	"	"
Caucasian (%)	11 (69)	10 (50)		"	"
African-American (%)	1 (6)	6 (30)		"	"
Asian (%)	2 (13)	0 (0)		"	"
Multiracial (%)	2 (13)	4 (20)		"	"
INCOME			0.525	"	"
<$20k (%)	6 (38)	5 (25)		"	"
$20-40k (%)	3 (19)	7 (35)		"	"
>$40k (%)	6 (38)	8 (40)		"	"
Unknown	1 (6)	0 (0)		"	"
EDUCATION			0.101	"	"
<=12 yrs (%)	0 (0)	4 (20)		"	"
13-16 yrs (%)	10 (63)	8 (40)		"	"
>16 yrs (%)	6 (38)	6 (30)		"	"
Unknown	0 (0)	2 (10)		"	"
EMPLOYMENT			0.122	"	"
Employed (%)	14 (88)	13 (65)		"	"
Unemployed (%)	2 (13)	7 (35)		"	"
HISTORY OF SUBSTANCE ABUSE			0.306	"	"
Yes (%)	2 (13)	5 (25)		"	"
No (%)	14 (88)	15 (75)		"	"
HAM-D,	1.19 (1.38)	All MDD: 21.2 (2.5)	<.001		
mean (SD)		MDD-only:21.6 (2.8)		MDD-only vs HC	<.001
		MDD+PTSD:20.8 (2.3)		MDD+PTSD vs HC	<.001
				MDD-only vs MDD+PTSD	0.673
HAM-A,	2.06 (2.27)	All MDD: 17.7 (5.6)	<.001		
mean (SD)		MDD-only:14.7 (5.2)		MDD-only vs HC	<.001
		MDD+PTSD:21.8 (3.0)		MDD+PTSD vs HC	<.001
				MDD-only vs MDD+PTSD	0.002
CTQ,	40.4 (9.5)	All MDD: 69.0 (23.6)	<.001		
mean (SD)		MDD-only:57.7 (10.8)		MDD-only vs HC	<.001
		MDD+PTSD:82.8 (28.1)		MDD+PTSD vs HC	<.001
				MDD-only vs MDD+PTSD	0.033

CTQ: Childhood trauma questionnaire; HAM-A: Hamilton Anxiety Rating Scale; HAM-D: Hamilton Depression Rating Scale; HC: Healthy control; MDD-only: Major depressive disorder; MDD+PTSD: Primary posttraumatic stress disorder comorbid with major depressive disorder

doi: 10.1371/journal.pone.0078292.t001

discounting over losses (δ * g * l). As illustrated in Figure 2A, depressed subjects show significantly steeper discounting for gains than HC. This pattern was observed for early delays (β * g: parameter estimate: 0.876, $p < 0.005$), as well as late delays over gains (δ * g: parameter estimate: 0.054, $p < 0.005$). No significant differences were observed for discounting over long delays for losses. Taken together, these results indicate that subjects in a current major depressive episode more steeply discount gains over time than HC.

Moderating effects of PTSD on intertemporal decision-making in depressed subjects. Figures 2B and 2C illustrate the moderating effect of PTSD within depressed subjects on intertemporal choice, based on the best-fitting 9-parameter model comparing the three groups. Table 3 shows parameter estimates for individual groups that reflect slopes for early discounting for delays up to 1 year (β), and late discounting for both gains (δ) and losses (δ * l) for delays greater than 1 year. Of greatest significance to the current analyses are parameter estimates reflective of comparisons between the depressed and healthy control groups, including group effects on (1) early discounting (β * g), (2) late discounting over gains (δ * g) and (3) late discounting over losses (δ * g * l). Quasi-hyperbolic discounting functions for all groups are illustrated in Figure 2B, while discounting functions together with mean delay equivalents from the bisection method for individual groups are shown in Figure 2C and 2D. Overall, results indicate that PTSD

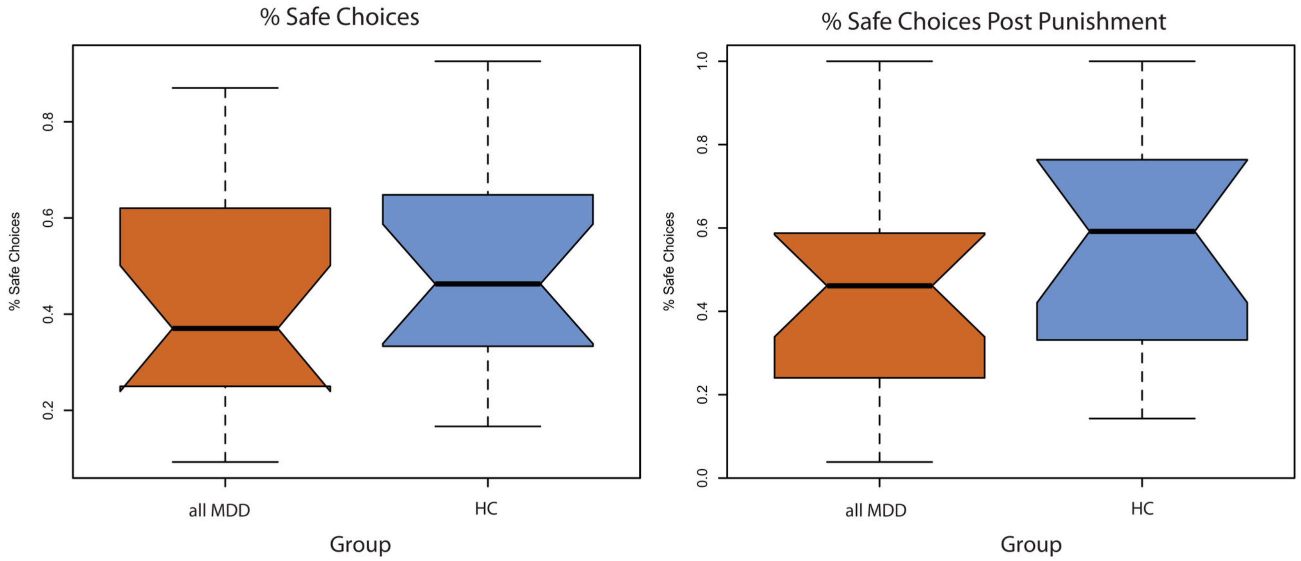

Figure 1. Group differences in choice frequencies during risky decision-making. HC: Healthy controls; MDD: Major depressive disorder.
Notched box plots illustrate choice frequencies as a function of group (all MDD and HC). Notches reflect confidence intervals around the median (median +/- (1.57 x IQR/√n), and, in case of no overlap, indicate significant differences between medians. No differences between all MDD and HC were observed for median choice frequencies over the safe option (20 points) overall, as well as immediately following a punished trial.
doi: 10.1371/journal.pone.0078292.g001

functions as a moderating factor within depressed subjects, such that a PTSD diagnosis is associated with a significant change in temporal discounting over long term losses relative to MDD-only subjects.

Specifically, the groups demonstrate different early discounting slopes as indicated by the significant interaction term $\beta * g$, such that MDD-only and MDD+PTSD subjects show steeper slopes than HC reflective of greater temporal discounting for both gain and loss choice options in the immediate future (for D ≤1 year; HC vs. MDD-only: $p < 0.05$, HC vs. MDD+PTSD: $p < 0.05$). For intertemporal choices involving potential gains over delays of >1-10 years, MDD-only and MDD+PTSD subjects continue to show significantly steeper slopes than HC as indicated by the significant interaction term (δ* g, for D >1 year; HC vs. MDD-only: $p < 0.05$, HC vs. MDD+PTSD: $p < 0.05$). These results indicate that both depressed groups value long-term future rewards significantly less relative to immediate outcomes than HC. In the domain of losses, MDD-only subjects do not adjust their discounting functions like the other two groups (difference between gain and losses in HC: $p < 0.001$, in MDD+PTSD: $p = 0.009$), indicating that, compared to MDD+PTSD subjects and HC, MDD-only subjects have greater preference for larger later losses over smaller immediate losses. Importantly, choice patterns of MDD+PTSD subjects differ significantly from the other two groups in that they show a significantly greater change from short- to long-term outcomes as indicated by the significant interaction term (δ* g * I). Specifically, while choice patterns of MDD+PTSD subjects are similar to MDD-only subjects for outcomes after short-delays and gains after >1-10 year delays, their choice patterns look more like HC for losses over the >1-10 year delay period. These results indicate that the effect of loss framing was greatest on choice patterns of MDD+PTSD subjects relative to the MDD-only and HC (HC vs. MDD+PTSD: $p < 0.05$, MDD-only vs MDD+PTSD: $p < 0.05$).

Decision-making Questionnaires

Table 4 summarizes the results of the BIS and FDMQ questionnaires. Depressed subjects reported significantly more overall impulsivity than HC subjects, as well as Attentional and Non-Planning impulsivity factors. There were no differences between MDD-only and MDD+PTSD subjects on any impulsivity measure.

On the FDMQ, depressed subjects scored higher than HC on Hypervigilance, Procrastination and Buck-Passing factors. HC scored significantly higher than depressed subjects on Vigilance. Post hoc testing of Vigilance scores did not find differences between MDD-only and HC, but MDD+PTSD subjects were significantly lower than HC.

Discussion

In this study of subjects in a current major depressive episode and HC subjects, we found substantial differences in temporal discounting between healthy and depressed subjects. Furthermore, the effect of PTSD modified the discounting for losses observed among subjects in a current major depressive episode. These differences in discounting did not derive from

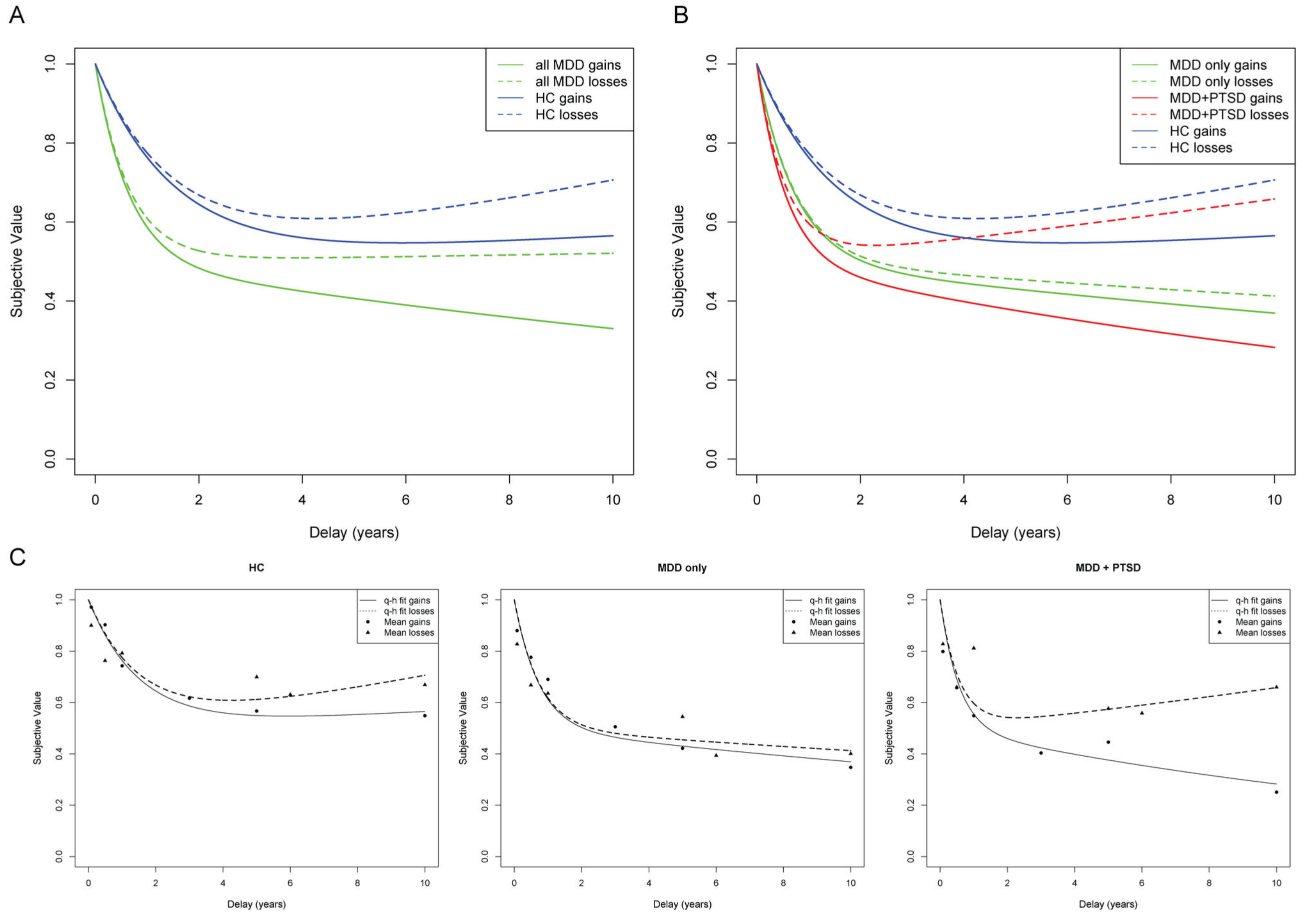

Figure 2. Quasi-hyperbolic discounting functions across groups. HC: Healthy controls; MDD-only: Major depressive disorder without PTSD; MDD+PTSD: Primary posttraumatic stress disorder comorbid with major depressive disorder.Temporal discounting reflects a decrease in subjective value as the time to the outcome increases. Subjective value is shown in all figures as a fraction of the immediate outcome. (A) Steeper discounting slopes for all MDD subjects relative to HC were found for short-term and long-term gains, but not for long-term losses. (B) PTSD significantly modulates temporal discounting over long-term losses, as demonstrated via significant differences in the slope for later losses between MDD only and MDD+PTSD subjects. (C) To illustrate model fits for each group, quasi-hyperbolic discounting functions are shown for each group separately together with means of subjective values obtained from the bisection method.

doi: 10.1371/journal.pone.0078292.g002

different risk attitudes, as we observed no differences between the groups on the level of risk taking, nor in the sensitivity to punishment on the Risky Gains Task.

The most intriguing result of this study was the dissociation between depressed subjects with and without PTSD in the area of long-term (>1 year) discounting of losses. Both groups showed the expected steep discounting for gains in both short-term and long-term time frames, indicating a greater preference than controls to take smaller immediate outcomes rather than await a later, larger gain. In the domain of losses, however, significant differences between the depressed groups were observed. Although both groups of depressed subjects showed steeper discounting than healthy subjects for losses delayed up to a year, PTSD subjects demonstrate a stark and significant recovery from steep discounting for loss outcomes beyond one year. This finding was contrary to our hypothesis that the PTSD symptom of a foreshortened future would drive steeper discounting over both losses and gains.

The shallower discounting of future losses in the healthy controls and MDD+PTSD versus the MDD-only group suggests the MDD-only group did not experience disutility in dreading a known long-term loss, which others have observed in healthy subjects [54]. The MDD-only subjects clearly preferred to accept a larger later loss to avoid immediate losses, perhaps stemming from profound pessimism about the future. Alternatively, the aversion of experiencing an immediate loss relative to that of a delayed loss may have been exacerbated by the presence of an already aversive mood state. According to this hypothesis, current depressed mood state creates an affective context that leads to choice distortions relative to

Table 2. Comparison between all depressed (MDD-only and MDD+PTSD) and healthy control (HC) subjects.

Parameters	Estimate (bias)	SEM	t	p	95% CI $_{boot}$	
					Lower Limit	Upper Limit
**Early Discounting (β)**						
HC	0.664 (0.007)	0.147	4.939	< 0.001	0.4133	0.9922
MDD	1.540 (-0.049)	0.372	6.148	< 0.001	1.074	2.479
**Group differences in early discounting (β* g)**						
HC vs. MDD	0.876 (0.048)	0.398	3.081	0.002	0.3018	1.7999
**Late Discounting over gains (δ)**						
HC	-0.012 (0.001)	0.012	-1.155	0.249	-0.0338	0.0126
MDD	0.042 (0.001)	0.013	2.999	0.003	0.0165	0.0681
**Late Discounting over losses (δ* l)**						
HC	-0.022 (-0.000)	0.014	-1.739	0.083	-0.0501	0.0044
MDD	-0.046 (-0.000)	0.016	-2.798	0.005	-0.0773	-0.0138
**Group differences in late discounting over gains (δ* g)**						
HC vs. MDD	0.054 (-0.000)	0.018	3.088	0.002	0.0187	0.0883
**Group differences in late discounting over losses (δ* g* l)**						
HC vs. MDD	-0.023 (0.000)	0.021	-1.123	0.262	-0.0648	0.0181

Results from the best fitting quasi-hyperbolic model and bootstrap analysis showing parameter estimates for each group and group differences in early and late discounting over gains and losses.

β: early discounting, δ: late discounting, g: effect of group on discounting slope, l: effect of loss on discounting slope. Parameter estimates are shown together with their estimated bias based on bootstrap analyses (in parentheses), respective standard errors (SEM), t statistic, p values, and 95% confidence intervals from confirmatory nonparametric bootstrap analyses.

Parameter estimates reflect quasi-hyperbolic intertemporal choice functions for each group (β, δ and, δ * l). Interactions with the group dummy (g) reflect differences in slopes between groups for delays smaller than one year (β* g), as well as delays greater than one year in the domain of gains (δ* g) and losses (δ* g * l).

doi: 10.1371/journal.pone.0078292.t002

healthy controls and MDD+PTSD subjects, who both preferred to take a smaller loss now rather than incur a larger later loss.

This willingness of MDD+PTSD subjects to accept an immediate loss to avoid a larger later one may reflect an anxious attitude about negative future events. For example, victims of domestic violence who have PTSD often continue to live with their abusers for years before moving out to a shelter or some other source of refuge. Although many practical aspects may affect such decision-making, one conceptualization consistent with this study's findings is that accepting the pain of physical abuse now is more acceptable to depressed PTSD patients than larger perceived long-term losses incurred from being dispossessed of one's home and belongings. It is also possible that the disutility associated with dreading a long-term high cost is greater for anxiously depressed PTSD patients than MDD patients without PTSD. Interestingly, the negative experience of dread of future pain is processed partly through the anterior cingulate cortex [54], a region showing hypofunction in PTSD patients exposed to threat.

The finding of reduced discounting of losses over the >1-10 year frame suggests that the presence of anxiety associated with PTSD in the setting of a major depressive episode may serve protective functions against overly pessimistic-based choices in MDD-only subjects. Although the presence of anxiety in patients suffering from MDD predicts poor response to antidepressant treatments in terms of symptom reduction [55], real-world decision-making may be improved by this countervailing force. Further research on discounting in patients with anxiety disorders without MDD, and in MDD patients with other anxiety disorders (particularly generalized anxiety disorder) are needed to clarify this potentially protective role of anxiety.

Together, these results inform future research investigating the underlying affective and cognitive processes, as well as related neural mechanisms, of the observed choice distortions in patients with MDD. Research in neuroeconomics has made considerable progress in identifying the neural mechanisms involved in intertemporal decision-making. From a number of recent studies it has become apparent that intertemporal choice is a complex behavior that engages multiple affective and cognitive processes that are mediated by distinct but interacting neural networks [29,47,56-61]. These processes include: (1) choice conflict, which requires a resolution of conflict between two or more competing choice options to compute a decision. Choice conflict has repeatedly been associated with activity in anterior cingulate cortex (ACC) [62]; (2) valuation, which informs choice conflict by assigning subjective value to different choice options. A large body of evidence implicates two highly interconnected brain areas, namely the ventral striatum (VS) and ventromedial prefrontal cortex (vmPFC) in value computations [63]; (3) self-control, which needs to be engaged in situations in which subjective valuation alone leads to an outcome that is in conflict with long-term goals in order to ensure optimal decision-making. Two recent studies have implicated the dorsolateral prefrontal cortex (dlPFC) in self-control during intertemporal choice [59,60]; and (4) prospection, which is the ability to engage in

Table 3. Comparison between depressed subjects with MDD only, versus those with primary PTSD and comorbid MDDand healthy controls (HC).

Parameters	Estimate	SEM	t	p	95% CI $_{boot}$	
	(bias)				Lower Limit	Upper Limit
**Early Discounting (β)**						
HC	0.664 (0.007)	0.134	4.974	<0.001	0.4101	0.9967
MDD only	1.370 (0.053)	0.297	4.613	<0.001	0.862	2.374
MDD + PTSD	1.800 (0.498)	0.442	4.072	<0.001	1.002	8.652
**Group differences in early discounting (β* g)**						
HC vs. MDD only	0.706 (0.005)	0.326	2.168	0.031	0.1028	1.6968
HC vs. MDD + PTSD	1.136 (0.490)	0.462	2.460	0.014	0.265	8.065
MDD only vs. MDD + PTSD	0.430 (0.445)	0.533	0.807	0.420	-0.7816	7.0652
**Late Discounting over gains (δ)**						
HC	-0.012 (0.001)	0.013	1.752	0.081	-0.0336	0.0123
MDD only	0.030 (0.001)	0.022	0.507	0.612	0.0006	0.0632
MDD + PTSD	0.057 (0.002)	0.025	3.332	<0.001	0.0126	0.1034
**Late Discounting over losses (δ* l)**						
HC	-0.034 (-0.001)	0.008	-4.431	<0.001	-0.0502	0.004
MDD only	0.019 (-0.000)	0.014	1.369	0.172	-0.0558	0.0352
MDD + PTSD	-0.027 (-0.002)	0.011	-2.613	0.009	-0.1326	-0.0353
**Group differences in late discounting over gains (δ* g)**						
HC vs. MDD only	0.042 (-0.0002)	0.020	2.132	0.034	0.0035	0.0817
HC vs. MDD + PTSD	0.069 (0.0005)	0.025	2.726	0.007	0.0194	0.1233
MDD only vs. MDD + PTSD	0.027 (0.0007)	0.029	0.932	0.352	-0.0285	0.0835
**Group differences in late discounting over losses (δ* g* l)**						
HC vs. MDD only	0.011 (0.0005)	0.025	0.441	0.659	-0.0417	0.0652
HC vs. MDD + PTSD	-0.062 (-0.001)	0.028	-2.191	0.029	-0.1179	-0.0069
MDD only vs. MDD + PTSD	-0.073 (0.001)	0.034	-2.189	0.029	-0.1400	-0.0071

Results from best fitting quasi-hyperbolic model and bootstrap analysis showing parameter estimates for each group and group differences in early and late discounting over gains and losses.

β: early discounting, δ: late discounting, g: effect of group on discounting slope, l: effect of loss on discounting slope. Parameter estimates are shown together with their estimated bias based on bootstrap analyses (in brackets), respective standard errors (SEM), t statistic, p values, and 95% confidence intervals from confirmatory nonparametric bootstrap analyses.

Parameter estimates reflect quasi-hyperbolic intertemporal choice functions for each group (β, δ and, δ * l). Interactions with the group dummy (g) reflect differences in slopes between groups for delays smaller than one year (β* g), as well as delays greater than one year in the domain of gains (δ* g) and losses (δ* g * l).

doi: 10.1371/journal.pone.0078292.t003

episodic future thought and is required to form mental representations of future goals. A recent study demonstrated that the ability to engage in prospection significantly modulated impulsivity during intertemporal choice, which was associated with strengthened functional connectivity between ACC and hippocampus [61].

The choice distortions observed in patients with MDD in the current investigation are likely due to changes in multiple interacting brain systems. Primary among those are systems known to be altered by depression and anxiety, including (1) valuation, which is expected to be particularly distorted in the presence of anhedonia, a common sympton of MDD; (2) choice conflict, which is expected to be biased in the presence of anxiety about aversive events in the future, a common symptom in PTSD; and (3) the ability to simulate future outcomes, which our results suggest to be attenuated in patients with MDD only. Interestingly, the functional connectivity between ACC and hippocampus has been associated with reductions in impulsive intertemporal decision-making [61], both of which are regions whose structural and functional integrity is affected by MDD.

Some have argued that intertemporal choice behavior may be confounded by risk attitude because delays entail an inherent risk that the future event may never occur [37,63]. Furthermore, the processing of decisions involving risk and delayed consequences engages partially overlapping brain networks, including DLPFC, OFC, ventral striatum and anterior insula [49,64-67]. It is therefore important to note that we did not find significant differences between depressed and HC groups in their propensity to take risks. These results indicate that, against concerns raised by economists, our current findings on intertemporal decision-making are likely not driven by group differences in risk attitudes. Future investigations of risk between depressed and control subjects should identify the underlying mechanisms leading to the observed differences. Such investigations may benefit from using more established neuroeconomic probes of risk attitude, which typically sample

Table 4. Depressed versus healthy control subjects self-reported impulsivity and decision-making styles.

Characteristic	HC (N=16)	Depressed (N=20)	p-value	Pair-wise Comparison	p-value
**BIS**					
Total,	49.2 (7.0)	All MDD: 64.5 (14.2)	0.001		
mean (SD)		MDD-only: 63.0 (12.0)		MDD-only vs HC	0.004
		MDD+PTSD: 66.3 (16.5)		MDD+PTSD vs HC	0.008
				MDD-only vs MDD+PTSD	0.518
Attentional Impulsivity,	12.4 (2.3)	All MDD: 17.4 (4.9)	0.001		
mean (SD)		MDD-only: 16.8 (3.3)		MDD-only vs HC	0.001
		MDD+PTSD: 18.0 (6.5)		MDD+PTSD vs HC	0.021
				MDD-only vs MDD+PTSD	0.878
Motor Impulsivity, mean (SD)	18.9 (2.5)	All MDD: 21.2 (5.6)	0.345	N/A	N/A
Non-Planning Impulsivity,	17.8 (4.1)	All MDD: 26.0 (5.9)	<.001		
mean (SD)		MDD-only: 25.7 (5.6)		MDD-only vs HC	0.003
		MDD+PTSD: 26.2 (6.6)		MDD+PTSD vs HC	0.004
				MDD-only vs MDD+PTSD	0.939
**FDMQ**					
Vigilance,	11.1 (1.5)	All MDD: 10.6 (2.2)	0.021		
mean (SD)		MDD-only: 9.9 (2.2)		MDD-only vs HC	0.102
		MDD+PTSD: 9.1 (2.3)		MDD+PTSD vs HC	0.021
				MDD-only vs MDD+PTSD	0.437
Hypervigilance,	2.2 (1.6)	All MDD: 5.5 (1.9)	<.001		
mean (SD)		MDD-only: 5.3 (1.8)		MDD-only vs HC	0.001
		MDD+PTSD: 5.7 (2.0)		MDD+PTSD vs HC	0.001
				MDD-only vs MDD+PTSD	0.562
Buck-Passing,	2.8 (2.5)	All MDD: 6.4 (3.8)	0.008		
mean (SD)		MDD-only: 6.0 (3.7)		MDD-only vs HC	0.036
		MDD+PTSD: 6.8 (4.0)		MDD+PTSD vs HC	0.019
				MDD-only vs MDD+PTSD	0.619
Procrastination,	1.8 (1.4)	All MDD: 5.4 (2.6)	<.001		
mean (SD)		MDD-only: 5.6 (2.7)		MDD-only vs HC	<.001
		MDD+PTSD: 5.0 (2.6)		MDD+PTSD vs HC	0.002
				MDD-only vs MDD+PTSD	0.513

BIS: Barratt Impulsivity Scale; HC: Healthy control; FDMQ: Flinders Decision-Making Questionnaire; MDD-only: Major depressive disorder; MDD+PTSD: Primary posttraumatic stress disorder comorbid with major depressive disorder

doi: 10.1371/journal.pone.0078292.t004

from a more extensive decision-space over multiple probabilities and payout magnitudes.

Our results both agree and conflict with the only prior study of intertemporal choice in depressed patients. Consistent with our results, Takahashi and colleagues [36] reported greater levels of impulsivity for near-future outcomes in depressed compared to healthy subjects, as well as similar discounting of distant-future gains and losses in depressed subjects. Importantly, their depressed patients showed almost no discounting of losses in the distant future, an effect that in our investigation was significantly modulated by the presence of PTSD. Despite the similar results for depressed subjects across the two investigations, we observe different choice patterns for long-term gains and losses in our healthy control subjects. Specifically, contrary to results from Takahashi and colleagues, healthy controls in our investigation showed minimal discounting of long-term future gains and losses. Potential contributors to the differing results include the differing intertemporal choice tasks and econometric models used, as well as the participant's culture (Western versus Japanese), which can have a significant impact on decision-relevant neural and cognitive mechanisms [68].

A potential limitation of this study is the use of hypothetical rewards. Specifically, artificial reward "points" formed the basis of motivation in both the Risky Gains and the Temporal Discounting tasks. Although real payoffs may increase salience and more strongly engage anticipatory emotions in decision-making, previous work suggests the use of hypothetical rewards does not lead to meaningfully different choices from those involving real outcomes [69,70]. Strengths of the study include the well-matched groups, and that all depressed subjects were suffering from moderate to severe depression, so failure to identify disturbances due to mild symptoms was avoided.

The results of the present study suggest that there are significant differences between healthy subjects and depressed patients in the way they make intertemporal decisions. Intertemporal decisions are ubiquitous in real life and have

been shown to significantly impact important facets of life, such as relationships, physical health and wealth [26]. The observed behavioral differences therefore may have important implications for treatment. For example, specific components of psychotherapeutic approaches for patients with major depression may benefit from modifications based on the degree to which a patient discounts future outcomes. Delay discounting tasks may have particular value in research on suicide, in that patients who most steeply discount future outcomes may be at heightened suicide risk. Coupling real-world choice behaviors by patients, such as credit card spending and saving habits, with laboratory measures of decision-making are needed to determine the clinical value of this line of research.

Finally, the differences we identified in choice behavior between depressed subjects with a primary diagnosis of PTSD versus those MDD subjects without PTSD suggest changes in the underlying neurobiological mechanisms involved in intertemporal choice [61]. Key networks that are likely to impact decision-making and have been shown to be affected by PTSD include the amygdala - prefrontal circuitry [22]. Future neuroimaging research is required to test the extent to which brain networks implicated in computations of important decision parameters are affected by the presence of MDD and PTSD.

Conclusion

Employing (neuro-)economic decision-making paradigms to investigate choice distortions in patients with psychiatric disorders can convey important insights into clinical features of mood and anxiety disorders. Anxiety or dread may lead depressed patients with a primary diagnosis of PTSD to make more conservative decisions about long-term losses that provide partial protection from negative consequences derived from the short-term outlook that drives decision-making among other patients with MDD.

Supporting Information

File S1. Text S1, Text S2, Figure S1, Tables S1-S6. Text S1. Description of Additional Questionnaires. Text S2. Model Selection. Figure S1. Frequencies of selecting the safe choice option during the risk task in all trials (A) and after punishment only (B) across three groups. The risk task was analyzed using both two (results reported in main paper) and three groups, in order to account for the moderating effects of comorbid PTSD. Results for the three-group analysis investigating the moderating effect of comorbid PTSD replicated findings for the two-group analysis. No difference between the groups were found for choice frequencies of safe option overall (A) and after punishment (B). Specifically, as illustrated in the box plots risky choice behavior did not differ between the HC, MDD-only and MDD+PTSD groups [Safe choice shown in A: X^2 (df = 2, N = 36) = 2.2973, p = 0.3171; Safe choice after punishment shown in B: X^2 (df = 2, N = 36) = 1.731, p = 0.421]. Table S1. Model comparison results across three commonly used discounting functions for the combined MDD analysis (see Text S2). The winning model is highlighted in bold. Table S2. Model comparison results across three commonly used discounting functions when stratifying the MDD group by comorbid PTSD diagnosis (see Text S2). The winning model is highlighted in bold. Table S3. Model comparison results across three commonly used discounting functions for control subjects (see Text S2). The winning model is highlighted in bold. Table S4. Model comparison results across three commonly used discounting functions for all MDD subjects (see Text S2). The winning model is highlighted in bold. Table S5. Model comparison results across three commonly used discounting functions for MDD only subjects (see Text S2). The winning model is highlighted in bold. Table S6. Model comparison results across three commonly used discounting functions for MDD+PTSD subjects (see Text S2). The winning model is highlighted in bold.
(PDF)

Acknowledgements

We would like to thank Thomas Epper for helpful discussion, and Benjamin Johnson for editing assistance.

Author Contributions

Conceived and designed the experiments: BWD. Performed the experiments: BM CV BWD. Analyzed the data: JBE BM MPP BWD. Contributed reagents/materials/analysis tools: MPP. Wrote the manuscript: BM JBE MPP BWD.

References

1. ESEMeD/MHEDEA 2000 Investigators (2004) Prevalence of mental disorders in Europe: results from the European Study of the Epidemiology of Mental Disorders (ESEMeD) project. Acta Psychiatr Scand 109 Suppl: 21-27. doi:10.1111/j.1600-0047.2004.00327.x.
2. Kessler RC, Berglund P, Demler O, Jin R, Merikangas KR et al. (2005) Lifetime prevalence and age-of-onset distributions of DSM-IV disorders in the National Comorbidity Survey Replication. Arch Gen Psychiatry 62: 593-602. doi:10.1001/archpsyc.62.6.593. PubMed: 15939837.
3. Henrique JB, Davidson RJ (2000) Decreased responsiveness to reward in depression. Cogn Emot 14: 711-724. doi: 10.1080/02699930050117684.
4. Pizzagalli DA, Iosifescu D, Hallett LA, Ratner KG, Fava M (2008) Reduced hedonic capacity in major depressive disorder: evidence from a probabilistic reward task. J Psychiatr Res 43: 76-87. doi:10.1016/j.jpsychires.2008.03.001. PubMed: 18433774.
5. Slade T, Watson D (2006) The structure of common DSM-IV and ICD-10 mental disorders in the Australian general population. Psychol Med 36: 1593-1600. doi:10.1017/S0033291706008452. PubMed: 16882356.
6. Blanchard EB, Buckley TC, Hickling EJ, Taylor AE (1998) Posttraumatic stress disorder and comorbid major depression: is the correlation an illusion? J Anxiety Disord 12: 21-37. doi:10.1016/S0887-6185(97)00047-9. PubMed: 9549607.
7. Grant DM, Beck G, Marques L, Palyo SA, Clapp JD (2008) The structure of distress following trauma: posttraumatic stress disorder, major depressive disorder and generalized anxiety disorder. J Abnorm Psychol 117: 662-672. doi:10.1037/a0012591. PubMed: 18729617.
8. Kessler RC (2000) Posttraumatic Stress Disorder: The burden to the individual and to society. J Clin Psychiatry 61 Suppl: 4-12. PubMed: 10761674.

9. Cella M, Dymond S, Cooper A (2010) Impaired flexible decision-making in major depressive disorder. J Affect Disord 124: 207-210. doi:10.1016/j.jad.2009.11.013. PubMed: 20004023.
10. Destoop M, Schrijvers D, De Grave C, Sabbe B, De Bruijn ER (2012) Better to give than to take? Interactive social decision-making in severe major depressive disorder. J Affect Disord 137: 98–105. doi:10.1016/j.jad.2011.12.010. PubMed: 22240086.
11. Kovacs M, Rottenberg J, George C (2009) Maladaptive mood repair responses distinguish young adults with early-onset depressive disorders and predict future depression outcomes. Psychol Med 39: 1841-1854. doi:10.1017/S0033291709005789. PubMed: 19379534.
12. Must A, Juhász A, Rimanóczy A, Szabó Z, Kéri S et al. (2007) Major depressive disorder, serotonin transporter, and personality traits: why patients use suboptimal decision-making strategies? J Affect Disord 103: 273-276. doi:10.1016/j.jad.2007.02.001. PubMed: 17382402.
13. Kroes MCW, Rugg MD, Whalley MG, Brewin CR (2011) Structural brain abnormalities common to posttraumatic stress disorder and depression. J Psychiatry Neurosci 3: 256-265. PubMed: 21418787.
14. Du MY, Wu QZ, Yue Q, Li J, Liao Y et al. (2012) Voxelwise meta-analysis of gray matter reduction in major depressive disorder. Prog Neuropsychopharmacol Biol Psychiatry 36: 11-16. doi:10.1016/j.pnpbp.2011.09.014. PubMed: 22001316.
15. Kühn S, Gallinat J (2012) Gray matter correlates of posttraumatic stress disorder: a quantitative meta-analysis. Biol Psychiatry; epub ahead of print. PubMed: 22840760.
16. Greicius MD, Flores BH, Menon V, Glover GH, Solvason HB et al. (2007) Resting state connectivity in major depression : abnormally increased contributions form subgenual cingulate cortex and thalamus. Biol Psychiatry 62: 429-437. doi:10.1016/j.biopsych.2006.09.020. PubMed: 17210143.
17. Mansouri FA, Tanaka K, Buckley MJ (2009) Conflict-induced behavioral adjustment: a clue to the executive functions of the prefrontal cortex. Nat Rev Neurosci 10: 141-152. doi:10.1038/nrn2538. PubMed: 19153577.
18. Salerian AJ, Altar CA (2012) The prefrontal cortex influence over subcortical and limbic regions governs antidepressant response by N=H/(M+R). Psychiatry Res 204: 1-12. doi:10.1016/j.pscychresns.2012.04.019. PubMed: 23022274.
19. LeDoux J (2007) The amygdala. Curr Biol 17: R868-R874. doi:10.1016/j.cub.2007.08.005. PubMed: 17956742.
20. Pessoa L (2009) How does emotion and motivation direct executive control? Trends Cogn Sci 13: 160-166. doi:10.1016/j.tics.2009.01.006. PubMed: 19285913.
21. Bremner JD, Staib LJ, Kaloupek D, Southwick SM, Soufer R et al. (1999) Neural correlates of exposure to traumatic pictures and sound in Vietnam combat veterans with and without posttraumatic stress disorder: a positron emission tomography study. Biol Psychiatry 45: 806-816. doi:10.1016/S0006-3223(98)00297-2. PubMed: 10202567.
22. Shin LM, Rauch SL, Pitman RK (2006) Amygdala, medial prefrontal cortex, and hippocampal function in PTSD. Ann N Y Acad Sci 107: 167-179. PubMed: 16891563.
23. Treadway MT, Zald DH (2011) Reconsidering anhedonia in depression: Lessons from translational neuroscience. Neurosci Biobehav Rev 35: 537-555. doi:10.1016/j.neubiorev.2010.06.006. PubMed: 20603146.
24. Elman I, Lowen S, Frederick BB, Chi W, Becerra L et al. (2009) Functional neuroimaging of reward circuitry responsivity to monetary gains and losses in posttraumatic stress disorder. Biol Psychiatry 66: 1083-1090. doi:10.1016/j.biopsych.2009.06.006. PubMed: 19640506.
25. Hasler G (2012) Can the neuroeconomics revolution revolutionize psychiatry? Neurosci Biobehav Rev 36: 64-78. doi:10.1016/j.neubiorev.2011.04.011. PubMed: 21550365.
26. Moffitt TE, Arseneault L, Belsky D, Dickson N, Hancox RJ et al. (2011) A gradient of childhood self-control predicts health, wealth, and public safety. Proc Natl Acad Sci U S A 108: 2693–2698. doi:10.1073/pnas.1010076108. PubMed: 21262822.
27. Ainslie G (1975) Specious reward: A behavioral theory of impulsiveness and impulse control. Psychol Bull 82: 463-496. doi:10.1037/h0076860. PubMed: 1099599.
28. Frederick S, Loewenstein G, O'Donoghue T (2002) Time discounting and time preference: a critical review. J Econ Lit 40: 351-401. doi:10.1257/002205102320161311.
29. McClure SM, Laibson DI, Loewenstein G, Cohen JD (2004) Separate neural systems value immediate and delayed monetary rewards. Science 306: 503-507. doi:10.1126/science.1100907. PubMed: 15486304.
30. Lowenstein G (1987) Anticipation and the valuation of delayed consumption. Econ J 97: 666-684. doi:10.2307/2232929.
31. Read D (2004) Intertemporal Choice. In: D KoehlerN Harvey. Blackwell Handbook of Judgment and Decision Making. Oxford: Blackwell. pp. 424–443.
32. Kirby KN, Petry NM, Bickel WK (1999) Heroin addicts have higher discount rates for delayed rewards than non-drug-using controls. J Exp Psychol Gen 128: 78-87. doi:10.1037/0096-3445.128.1.78. PubMed: 10100392.
33. Hamilton KR, Potenza MN (2012) Relations among delay discounting, addictions, and money mismanagement: Implications and future directions. Am J Drug Alcohol Abus 38: 30-42. doi:10.3109/00952990.2011.643978. PubMed: 22211535.
34. Lempert KM, Pizzagalli DA (2010) Delay discounting and future-directed thinking in anhedonic individuals. J Behav Ther Exp Psychiatry 41: 258-264. doi:10.1016/j.jbtep.2010.02.003. PubMed: 20219184.
35. Schweighofer N, Bertin M, Shishida K, Okamoto Y, Tanaka SC et al. (2008) Low-serotonin levels increase delayed reward discounting in humans. J Neurosci 28: 4528-4532. doi:10.1523/JNEUROSCI.4982-07.2008. PubMed: 18434531.
36. Takahashi T, Oono H, Inoue T, Boku S, Kako Y et al. (2008) Depressive patients are more impulsive and inconsistent in intertemporal choice behavior for monetary gain and loss than healthy subjects – an analysis based on Tsallis' statistics. Neuro Endocrinol Lett 29: 351-358. PubMed: 18580849.
37. Engelmann JB, Brooks AM (2009) Behavioral and neural effects of delays during intertemporal choice are independent of probability. J Neurosci 29: 6055-6057. doi:10.1523/JNEUROSCI.1037-09.2009. PubMed: 19439582.
38. Epper T, Fehr-Duda H, Bruhin A (2011) Viewing the future through a warped lens: Why uncertainty generates hyperbolic discounting. J Risk Uncertainty 43: 169–203. doi:10.1007/s11166-011-9129-x.
39. First MB, Spitzer RL, Gibbon M, Williams JBW (1995) Structured Clinical Interview for DSM-IV Axis I Disorders-Patient Edition (SCID-I/P, Version 2.0). New York: Biometrics Research Department, New York State Psychiatric Institute.
40. Hamilton M (1960) A rating scale for depression. J Neurol Neurosurg, Psychiatry 23: 56-62. doi:10.1136/jnnp.23.1.56.
41. Hamilton M (1959) The assessment of anxiety states by rating. Br J Med Psychol 32: 50–55. doi:10.1111/j.2044-8341.1959.tb00467.x. PubMed: 13638508.
42. Bernstein DP, Fink L (1998) Childhood trauma questionnaire manual. San Antonio: Psychological Corporation.
43. Patton JH, Stanford MS, Barratt ES (1995) Factor structure of the Barratt Impulsiveness Scale. J Clin Psychol 51: 768-774. doi:10.1002/1097-4679(199511)51:6. PubMed: 8778124.
44. Mann L, Burnett PC, Radford M, Ford S (1997) The Melbourne decision making questionnaire: an instrument measure patterns for coping with decisional conflict. J Behav Decis Mak 10: 1-19. doi:10.1002/(SICI)1099-0771(199703)10:1.
45. Leland DS, Paulus MP (2005) Increased risk-taking decision-making but not altered response to punishment in stimulant-using young adults. Drug Alcohol Depend 78: 83-90. doi:10.1016/j.drugalcdep.2004.10.001. PubMed: 15769561.
46. Wittmann M, Leland DS, Paulus MP (2007) Time and decision making: differential contribution of the posterior insular cortex and the striatum during a delay discounting task. Exp Brain Res 179: 643-653. doi:10.1007/s00221-006-0822-y. PubMed: 17216152.
47. Kable JW, Glimcher PW (2007) The neural correlates of subjective value during intertemporal choice. Nat Neurosci 10: 1625-1633. doi:10.1038/nn2007. PubMed: 17982449.
48. Berns GS, Laibson D, Loewenstein G (2007) Intertemporal choice – toward an integrative framework. Trends Cogn Sci 11: 482-488. doi:10.1016/j.tics.2007.08.011. PubMed: 17980645.
49. Engelmann JB, Moore S, Capra M, Berns GS (2012) Differential neurobiological effects of expert advice on risky choice in adolescents and adults. Soc Cogn Affect Neurosci 7: 557-567. doi:10.1093/scan/nss050. PubMed: 22563008.
50. Burghart DR, Glimcher PW, Lazzaro SC (in press) An expected utility maximizer walks into a bar... J. J Risk Uncertainty.
51. Camerer C, Ho TH (1999) Experience-weighted attraction learning in normal form games. Econometrica 67: 827-874. doi:10.1111/1468-0262.00054.
52. Tymula A, Rosenberg Belmaker LA, Roy AK, Ruderman L, Manson K et al. (2012) Adolescents' risk-taking behavior is driven by tolerance to ambiguity. Proc Natl Acad Sci USA 109: 17135–17140. doi:10.1073/pnas.1207144109. PubMed: 23027965.
53. Davison AC, Hinkley DV (1997) Bootstrap Methods and their Application. Cambridge: Cambridge University Press.

54. Berns GS, Chappelow J, Cekic M, Zink CF, Pagnoni G et al. (2006) Neurobiological substrates of dread. Science 312: 754-758. doi: 10.1126/science.1123721. PubMed: 16675703.
55. Fava M, Rush AJ, Alpert JE, Balasubramani GK, Wisniewski SR et al. (2008) Difference in treatment outcome in outpatients with anxious versus non-anxious depression: a STAR*D report. Am J Psychiatry 165: 342-351. doi:10.1176/appi.ajp.2007.06111868. PubMed: 18172020.
56. Tanaka SC, Doya K, Okada G, Ueda K, Okamoto Y et al. (2009) Prediction of immediate and future rewards differentially recruits cortico-basal ganglia loops. Nat Neurosci 7: 887-893. doi:10.1038/nrmicro2245. PubMed: 15235607.
57. McClure SM, Ericson KM, Laibson DI, Loewenstein G, Cohen JD (2007) Time discounting for primary rewards. J Neurosci 27: 5796–5804. doi:10.1523/JNEUROSCI.4246-06.2007. PubMed: 17522323.
58. Luhmann CC, Chun MM, Yi D-J, Lee D, Wang X-J (2008) Neural dissociation of delay and uncertainty in intertemporal choice. J Neurosci 28: 14459–14466. doi:10.1523/JNEUROSCI.5058-08.2008. PubMed: 19118180.
59. Hare TA, Camerer CF, Rangel A (2009) Self-control in decision-making involves modulation of the vmPFC valuation system. Science 324(5927): 646–648. doi:10.1126/science.1168450. PubMed: 19407204.
60. Figner B, Knoch D, Johnson EJ, Krosch AR, Lisanby SH et al. (2010) Lateral prefrontal cortex and self-control in intertemporal choice. Nat Neurosci 13: 538–539. doi:10.1038/nn.2516. PubMed: 20348919.
61. Peters J, Büchel C (2010) Episodic future thinking reduces reward delay discounting through an enhancement of prefrontal-mediotemporal interactions. Neuron 66: 138–148. doi:10.1016/j.neuron.2010.03.026. PubMed: 20399735.
62. Pochon J-B, Riis J, Sanfey AG, Nystrom LE, Cohen JD (2008) Functional imaging of decision conflict. J Neurosci 28: 3468–3473. doi:10.1523/JNEUROSCI.4195-07.2008. PubMed: 18367612.
63. Prelec D, Loewenstein G (1991) Decision making over time and under uncertainty: A common approach. Manag Sci 37: 770–786. doi:10.1287/mnsc.37.7.770.
64. Engelmann JB, Tamir D (2009) Individual differences in risk preference predict neural response during financial decision-making. Brain Res 1290: 28-51. doi:10.1016/j.brainres.2009.06.078. PubMed: 19576868.
65. Schonberg T, Fox CR, Poldrack RA (2011) Mind the gap: bridging economic and naturalistic risk-taking with cognitive neuroscience. Trends Cogn Sci 15: 11-19. doi:10.1016/j.tics.2010.10.002. PubMed: 21130018.
66. Mohr PN, Biele G, Heekeren HR (2010) Neural processing of risk. J Neurosci 30: 6613-6619. doi:10.1523/JNEUROSCI.0003-10.2010. PubMed: 20463224.
67. Singer T, Critchley HD, Preuschoff K (2009) A common role of insula in feelings, empathy and uncertainty. Trends Cogn Sci 13: 334–340. doi:10.1016/j.tics.2009.05.001. PubMed: 19643659.
68. Engelmann JB, Pogosyan M (2013) Emotion perception across cultures: the role of cognitive mechanisms. Front Psychol. 4: 118. PubMed: 23486743.
69. Kuhberger A, Schulte-Mecklenbeck M, Perner J (2002) Framing decisions: Hypothetical and real. Org Behav Hum Decision Processes 89: 1162-1175. doi:10.1016/S0749-5978(02)00021-3.
70. Madden GJ, Begotka AM, Raiff BR, Kastern LL (2003) Delay discounting of real and hypothetical rewards. Exp Clin Psychopharmacol 11: 139-145. doi:10.1037/1064-1297.11.2.139. PubMed: 12755458.
